# Identification of Unique Key miRNAs, TFs, and mRNAs in Virulent MTB Infection Macrophages by Network Analysis

**DOI:** 10.3390/ijms23010382

**Published:** 2021-12-29

**Authors:** Tingting Zhu, Han Liu, Li Su, Ali Dawood, Changmin Hu, Xi Chen, Huanchun Chen, Yingyu Chen, Aizhen Guo

**Affiliations:** 1 The State Key Laboratory of Agricultural Microbiology, Huazhong Agricultural University, Wuhan 430070, China; 553238808@webmail.hzau.edu.cn (T.Z.); liuhan18415@163.com (H.L.); suli513@webmail.hzau.edu.cn (L.S.); ali.dawood@vet.usc.edu.eg (A.D.); hcm@mail.hzau.edu.cn (C.H.); chenxi@mail.hzau.edu.cn (X.C.); chenhch@mail.hzau.edu.cn (H.C.); 2 College of Veterinary Medicine, Huazhong Agricultural University, Wuhan 430070, China; 3 Department of Medicine and Infectious Diseases, Faculty of Veterinary Medicine, University of Sadat City, Sadat 32511, Egypt; 4 Key Laboratory of Development of Veterinary Diagnostic Products, Ministry of Agriculture, Huazhong Agriculture University, Wuhan 430070, China; 5 Key Laboratory of Ruminant Bio-Products, Ministry of Agriculture, Huazhong Agriculture University, Wuhan 430070, China; 6 Hubei International Scientific and Technological Cooperation Base of Veterinary Epidemiology, Huazhong Agricultural University, Wuhan 430070, China; 7 International Research Center for Animal Disease, Ministry of Science and Technology, Huazhong Agricultural University, Wuhan 430070, China; 8 National Animal Tuberculosis Para-Reference Laboratory, Huazhong Agricultural University, Wuhan 430070, China

**Keywords:** microRNA, RNAseq, tuberculosis, TF-miRNA-mRNA, network

## Abstract

Although *Mycobacterium tuberculosis* (MTB) has existed for thousands of years, its immune escape mechanism remains obscure. Increasing evidence signifies that microRNAs (miRNAs) play pivotal roles in the progression of tuberculosis (TB). RNA sequencing was used to sequence miRNAs in human acute monocytic leukemia cells (THP-1) infected by the virulent MTB-1458 strain and the avirulent vaccine strain *Mycobacterium bovis* Bacillus Calmette-Guérin (BCG). Sets of differentially expressed miRNAs (DE-miRNAs) between MTB-1458/BCG-infected groups and uninfected groups were identified, among which 18 were differentially expressed only in the MTB-1458-infected THP-1 group. Then, 13 transcription factors (TFs) and 81 target genes of these 18 DE-miRNAs were matched. Gene Ontology classification as well as Kyoto Encyclopedia of Genes and Genomes pathway enrichment analysis showed that the candidate targets were predominantly involved in apoptotic-associated and interferon-γ-mediated signaling pathways. A TF-miRNA-mRNA interaction network was constructed to analyze the relationships among these 18 DE-miRNAs and their targets and TFs, as well as display the hub miRNAs, TFs, and target genes. Considering the degrees from network analysis and the reported functions, this study focused on the BHLHE40-*miR-378d*-*BHLHE40* regulation axis and confirmed that *BHLHE40* was a target of *miR-378d*. This cross-talk among DE-miRNAs, mRNAs, and TFs might be an important feature in TB, and the findings merited further study and provided new insights into immune defense and evasion underlying host-pathogen interactions.

## 1. Introduction

Tuberculosis (TB) is a zoonotic communicable disease caused by *Mycobacterium tuberculosis* complex (MTBC) and is still one of the deadliest diseases worldwide. According to the 2021 global TB report by the World Health Organization (WHO), an estimated 10.0 million people fell ill with TB and there were 1.5 million TB deaths in 2020 [1]. The WHO has developed its TB termination strategy in 2014, but there is still a lack of efficient detection and treatment methods. This is attributed to the complex pathogenic mechanism of *Mycobacterium tuberculosis* (MTB). *Mycobacterium bovis* (*M. bovis*) Bacillus Calmette-Guérin (BCG) is the only existing TB vaccine that can induce different immune responses compared to the virulent strain [2]. Thus, studying the different interaction mechanisms between avirulent BCG and virulent MTB with the host is an effective method to clarify the specific pathogenesis of virulent MTB.

microRNA (miRNA) is a kind of small single-stranded noncoding RNA, about 22 nucleotides, mainly located in the cytoplasm to manipulate posttranscription regulation through translation suppression and mRNA degradation [3]. Transcription factors (TFs) could bind to a specific DNA sequence to increase or block the recruitment of RNA polymerase to regulate gene expression [4]. Thus, TF, miRNA, and mRNA can form multiple feed-forward and feed-backward loops in host-pathogen interactions. Over the past decade, extensive studies have been conducted to explore the biomarkers and pathogenesis of TB by analyzing miRNA expression profiles. For example, *miR-155* is exploited as a marker that can distinguish between nonvaccinated and BCG-vaccinated cattle before and after *M. bovis* infection [5]. Circulating *miR-29a* is considered as a marker for detecting the active pulmonary TB infection based on microarray expression profiling analysis [6]. In addition, *miR-20b-5p*, *miR-381-3p*, and *miR-889* can affect the host’s antibacterial ability by regulating apoptosis, antigen presentation, and autophagy [7,8,9]. There are also many studies using network analysis to tap novel immune regulatory mechanisms during MTB infection. Whole transcriptome sequencing of pulmonary TB patients and healthy individuals has been used to create a structured circRNA–miRNA–mRNA network to seek functional noncoding RNAs [10]. Although a few studies have reported differentially expressed miRNA (DE-miRNA) expression characteristics from virulent or avirulent bacteria infected cells compared to uninfected cells [11,12], a systematic TF-miRNA-mRNA network analysis, which takes unique DE-miRNAs in the virulent MTB infected group as the key to provide new insights into host-pathogen interactions, has not been reported yet.

This research dissected the expression profiles of miRNAs in virulent MTB-1458-infected THP-1 cells or avirulent BCG-infected THP-1 cells and uninfected cells using miRNA sequencing (miRNAseq). Eighteen DE-miRNAs were found uniquely differentially expressed in MTB-1458-infected macrophages, and their potential targets and TFs were identified using both the computational prediction method and mRNA sequencing (mRNAseq). The construction of the TF-miRNA-mRNA network provided a good foundation to further distinguish the different pathogenic mechanisms and immune defense abilities during the interaction of BCG or MTB with the host.

## 2. Results

### 2.1. Expression Profiles of miRNAs in THP-1 Cells after Infection

**Figure 1:**
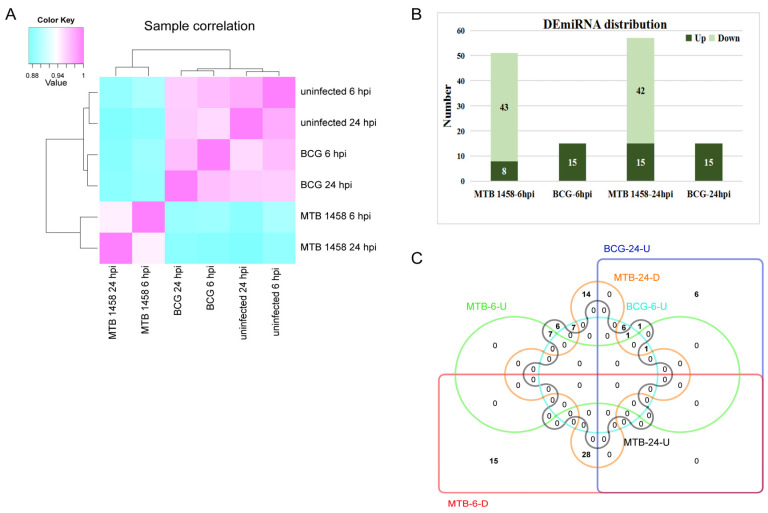
Expression profiles of miRNAs in THP-1 cells from different groups. (**A**) Sample correlation analysis. (**B**) DE-miRNA distribution of MTB-1458-6 hpi (MTB-1458-6 hpi vs. uninfected-6 hpi), BCG-6 hpi (BCG-6 hpi vs. uninfected-6 hpi), MTB-1458-24 hpi (MTB-1458-24 hpi vs. uninfected-24 hpi), and BCG-24 hpi (BCG-24 hpi vs. uninfected-24 hpi). (**C**) Venn diagram of BCG-6-U (upregulated miRNAs from BCG-6 hpi vs. uninfected-6 hpi), BCG-24-U (upregulated miRNAs from BCG-24 hpi vs. uninfected-24 hpi), MTB-6-U (upregulated miRNAs from MTB-1458-6 hpi vs. uninfected-6 hpi), MTB-6-D (downregulated miRNAs from MTB-1458-6 hpi vs. uninfected-6 hpi), MTB-24-U (upregulated miRNAs from MTB-1458-24 hpi vs. uninfected-24 hpi), and MTB-24-D (downregulated miRNAs from MTB-1458-24 hpi vs. uninfected-24 hpi).

miRNA expression profiles of MTB-1458-infected THP-1 cells or BCG-infected THP-1 cells at 6 and 24 h postinfection (hpi) were constructed. Uninfected THP-1 was used as the control. Generally, the expression profile of BCG-infected groups was more similar to uninfected groups than MTB-1458-infected groups (Figure 1A). Clean reads mapping against human mature miRNAs of miRBase results demonstrated that the proportions of miRNA reads in MTB-1458 groups were only 35% (6 hpi) and 33% (24 hpi), which were dramatically lower than in BCG and uninfected groups (all >70%; Table 1). These indicated that MTB-1458 induced a stronger miRNA expression profile variation and inhibited miRNA expression in the entire level.

A total of 1685 mature miRNAs were identified, including 234 novel miRNAs. For the 1451 known miRNAs, 92 were differentially expressed between MTB-1458-infected cells or BCG-infected cells and uninfected cells with a fold change of ≥2 or ≤0.5 (*p* ≤ 0.01; Figure S1). In detail, there were 22 DE-miRNAs between BCG-infected cells and uninfected cells, 15 upregulated DE-miRNAs at 6 and 15 upregulated DE-miRNAs at 24 hpi, and 8 miRNAs overlapped. There were 73 DE-miRNAs between MTB-1458-infected and uninfected cells. DE-miRNAs were 51 at 6 hpi, of which 43 miRNAs were downregulated and 8 were upregulated. At 24 hpi, DE-miRNAs were 57, of which 42 were downregulated and 15 were upregulated. Among them, 28 downregulated and 7 upregulated miRNAs were both differentially expressed at 6 and 24 hpi (Figure 1B,C).

In both the MTB-1458-infected group and the BCG-infected group, *miR-146a-3p* was upregulated at 6 hpi, while *miR-212-3p* and *miR-132-3p* were both upregulated at 24 hpi. Fourteen miRNAs at 6 hpi and thirteen miRNAs at 24 hpi were only differentially expressed after BCG infection, of which six miRNAs overlapped. Fifty miRNAs at 6 hpi and fifty-five miRNAs at 24 hpi were specifically regulated by MTB-1458, of which thirty-five miRNAs overlapped (Figure 1C; Table S1). Taken together, the results indicated that DE-miRNAs had little differences between 6 and 24 hpi in either BCG or MTB-1458 groups but showed a distinct difference within virulent strain-infected groups and avirulent strain-infected groups.

### 2.2. Screening miRNAs Only Differentially Expressed in MTB-1458 Groups

**Figure 2:**
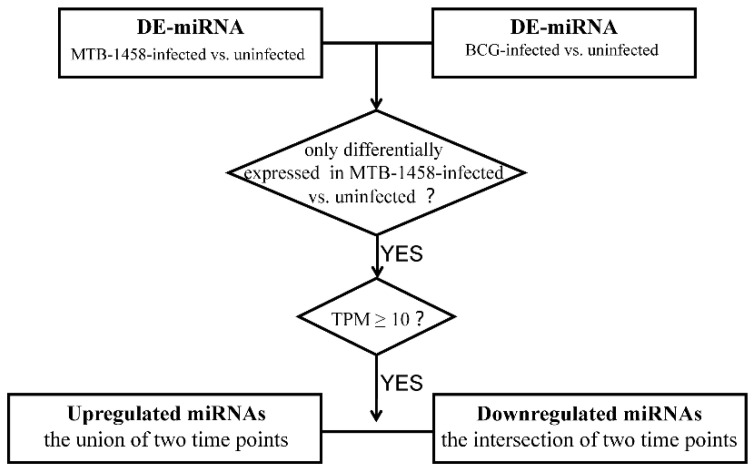
Screening flowchart. Flowchart of miRNA screening method for further analysis.

The screening was performed as displayed in the flowchart (Figure 2). Eighteen miRNAs only differentially expressed in MTB-1458 groups were selected for analysis. Among them, *miR-1290*, *miR-625-3p*, *miR-146a-5p*, *miR-96-5p*, and *miR-335-3p* were upregulated at 6 or 24 hpi. *miR-101-3p*, *miR-107*, *miR-1261*, *miR-140-3p*, *miR-146b-3p*, *miR-210*, *miR-3184-5p*, *miR-320b*, *miR-378c*, *miR-378d*, *miR-4286*, *miR-660-5p*, and *miR-874* were downregulated at both time points (Table 2).

### 2.3. Target and TF Prediction of Unique DE-miRNAs in MTB-1458 Groups

The potential targets of 18 selected DE-miRNAs in MTB-1458-infected groups were predicted by the miRDB and miRanda online databases, together with mRNAseq data. The potential TFs of the selected miRNAs were predicted by the TransmiR database. Fourteen potential targets and twelve candidate TFs were predicted for five upregulated DE-miRNAs, and sixty-seven potential targets and eleven candidate TFs were predicted for thirteen downregulated DE-miRNAs (Table 2). Excluding repeat TFs, 81 potential targets and 13 TFs were selected for further analysis.

### 2.4. Gene Ontology (GO) Classification and Kyoto Encyclopedia of Genes and Genomes (KEGG Pathways) Pathway Enrichment Analysis of the Candidate Targets

**Figure 3:**
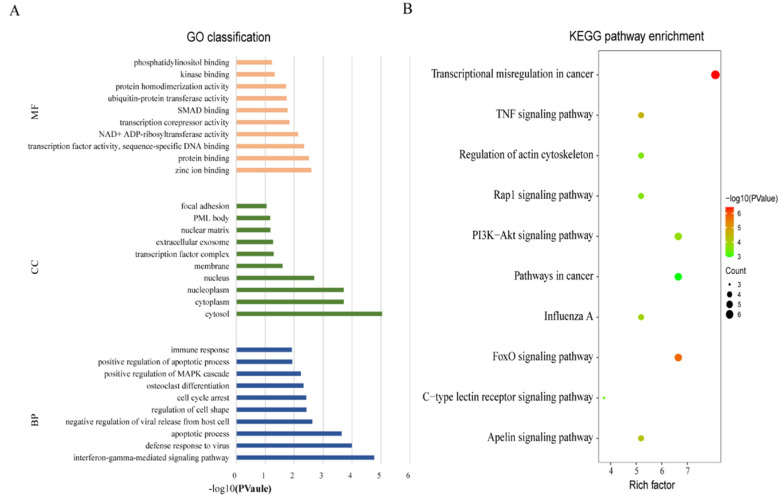
GO classification and KEGG pathway enrichment analysis of the candidate targets. (**A**) GO classification of the targets of unique DE-miRNAs in MTB-1458 groups were analyzed by DAVID 6.8 online database with default settings. The top 10 terms are displayed. (**B**) KEGG pathway enrichment analysis of the targets of unique DE-miRNAs in MTB-1458 groups was analyzed by KONAS 3.0 online database with default settings. The top 10 terms are displayed. Rich factor = enriched genes/total genes. The bubble size represents the number of enriched genes, and the color of the bubble represents −log10 (*p*-value).

To further understand the immune regulatory functions of the specific DE-miRNAs, the final candidate targets were subjected to GO classification and KEGG pathway enrichment analysis. The candidate targets were enriched in iron and protein, as well as DNA-binding-related molecular functions (MF) and interferon-γ (IFN-γ)-mediated signaling pathway and apoptotic-process-associated biological processes (BP), located in the cytosol of cellular component (CC; Figure 3A). For KEGG pathway enrichment analysis, 5 of the top 10 enriched pathways were associated with TB-related immune response, such as the Foxo signaling pathway (target genes: *PRKAG2*, *PLK3*, *TNFSF10*, *FBXO32*, and *SGK1*), tumor necrosis factor (TNF) signaling pathway (*TRAF1*, *IRF1*, *CSF1*, and *CEBPB*), phosphatidylinositol 3-kinase (PI3K-)/Akt signaling pathway (*PIK3R5*, *MYB*, *NGFR*, *SGK1*, and *CSF1*), Rap1 signaling pathway (*CTNNB1*, *CSF1*, *MRAS*, and *NGFR*), and C-type lectin receptor signaling pathway (*IRF1*, *PLK3*, and *MRAS*; Figure 3B). These candidate targets of DE-miRNAs might be pivotal mediators during MTB infection.

### 2.5. TF-miRNA-mRNA Network

**Figure 4:**
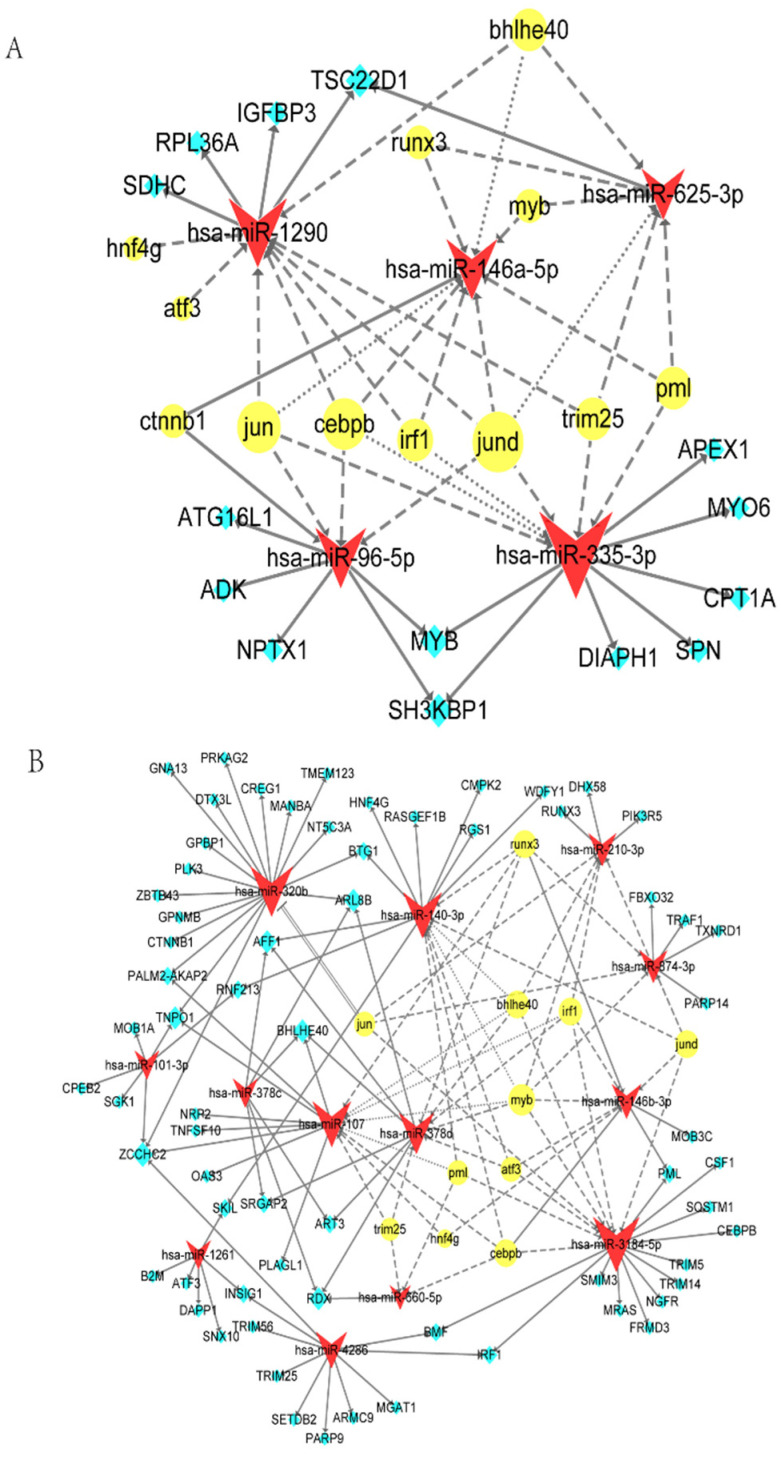
TF-miRNA-mRNA network. (**A**) Network analysis of 5 uniquely upregulated DE-miRNAs (red), 14 predicted targets (blue), and 12 predicted TFs (yellow) by Cytoscape. The size of the shape represents the degree of the nodes. Solid arrow, miRNA targets mRNA or TF activates miRNA; line dotted arrow, TF regulates miRNA; dotted arrow, TF regulates (feedback) miRNA. (**B**) Network analysis of 13 uniquely downregulated DE-miRNAs (red), 67 predicted targets (blue), and 11 predicted TFs (yellow) by Cytoscape. The size of the shape represents the degree of the nodes. Solid arrow, miRNA targets mRNA or TF activates miRNA; line dotted arrow, TF regulates miRNA; dotted arrow, TF regulates (feedback) miRNA; double solid, TF represses miRNA.

Combined with the above analysis, the complex regulatory network of miRNAs, mRNAs, and TFs was constructed and analyzed for key miRNAs, mRNAs, and TFs. In the five upregulated DE-miRNAs networks, *miR-335-3p* had the highest node degree of interactions, with seven targets and six TFs, followed by *miR-1290* with four targets and eight TFs, *miR-146-5p* with zero targets and nine TFs, and *miR-96-5p* with five targets and four TFs. *miR-625-3p* had the minimum regulation degree, with one target and six TFs. Three (*MYB*, *SH3KBP1*, and *TSC22D1*) of fourteen genes were targeted by two miRNAs, and others only had one associated miRNA. In TFs, JUND was associated with all five miRNAs, followed by CEBPB and JUN, each targeting four miRNAs. In all interactions, JUND-*miR-335-3p-MYB/SH3KBP1* was regarded as the key axis because of the highest degree of all three aspects in the network, which might affect the progress of MTB infection (Figure 4A).

In 13 downregulated DE-miRNAs network, *miR-3184-5p* had the highest degree of interactions, with 12 targets and 8 TFs, followed by *miR-320b* with 17 targets and 1 TF, *miR-107* with 8 targets and 8 TFs, and *miR-140-3p* with 9 targets and 7 TFs. Others had <16 connections. *miR-378c* and *miR-4286* had only targets but no TFs predicted because their relative data were absent in the database. In the 67 targets, *ZCCHC2* had the highest degree and was regulated by 4 miRNAs, followed by *TNPO1*, *BHLHE40*, *AFF1*, *ARL8B*, and *RDX*, all of them are targeted by 3 miRNAs. Eleven out of sixty-seven were regulated by two miRNAs, and fifty of them were targeted by only one miRNA. For TFs, the top ranking was MYB, which was linked to seven miRNAs. BHLHE40, CEBPB, IRF1, RUNX3, and JUND were correlated with five miRNAs. PML, ATF3, and JUN were involved with four miRNAs. In addition, TRIM25 and HNF4G were associated with three and two miRNAs, respectively (Figure 4B).

Considering the degrees from network analysis and reported functions, this study found that (i) MYB-*miR-3184-5p-BMF/IRF1/PML* had the highest degree of all TFs, miRNA, and mRNAs in the network. (ii) Three miRNA interactions, (IRF1-*miR-3184-5p-IRF1*, CEBPB-*miR-3184-5p-CEBPB*, and BHLHE40-*miR-107/miR-378d-BHLHE40*), presented the same targets and TFs, respectively, and the whole connection formed like a loop. (iii) Some targets, such as *ARL8B*, and TFs, such as MYB, IRF1, and CEBPB, participated in the TB immune-related BPs and had a higher degree in the network. However, these findings need to be validated in a series of subsequent experiments (Figure 4B).

### 2.6. Key DE-miRNA Validation by Quantitative Real-Time Polymerase Chain Reaction (qRT-PCR)

**Figure 5:**
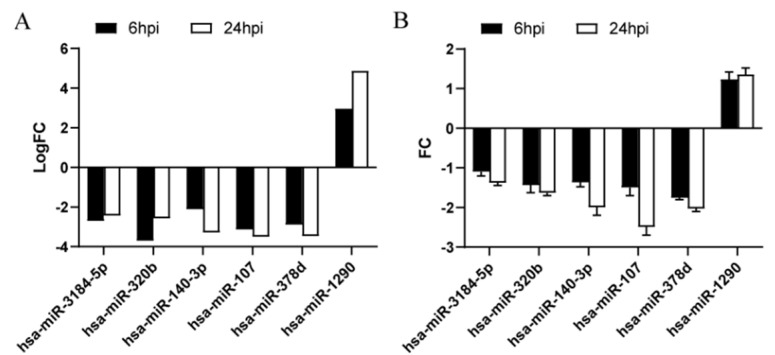
Key DE-miRNA validation by qRT-PCR. (**A**) logFC value of MTB-1458-infected cells vs. uninfected cells according to sequencing data. (**B**) THP-1 cells were infected with MTB-1458 at a MOI of 10 for 12 h. Cells were washed twice and a medium containing gentamycin (100 μg/mL) was added. At 6 and 24 hpi, total RNA was collected. The expression level of DE-miRNAs was measured by qRT-PCR and normalized with U6 as an internal reference.

Six DE-miRNAs (logFC > 2 at 6 and 24 hpi) were further validated by *qRT-PCR* according to the TF-miRNA-mRNA network analysis. *miR-3184-5p*, *miR-320b*, *miR-140-3p*, *miR-107*, and *miR-378d* were downregulated, whereas *miR-1290* was upregulated in MTB-1458-infected groups compared to uninfected groups, in accordance with sequencing data (Figure 5A,B).

### 2.7. BHLHE40-miR-378d-BHLHE40 Regulation Axis Verification

Since IRF1-*miR-3184-5p-IRF1*, CEBPB-*miR-3184-5p-CEBPB*, and BHLHE40-*miR-107/miR-378d-BHLHE40* were presented as a loop in the networks, the function of these three miRNAs and their targets was further analyzed. A previous study reported that BHLHE40 was an essential repressor of interleukin (IL)-10 during MTB infection and may have a potential function of the anti-MTB mice model [13]. Therefore, this study chose the BHLHE40-*miR-107/miR-378d-BHLHE40* regulation axis for further investigation.

**Figure 6:**
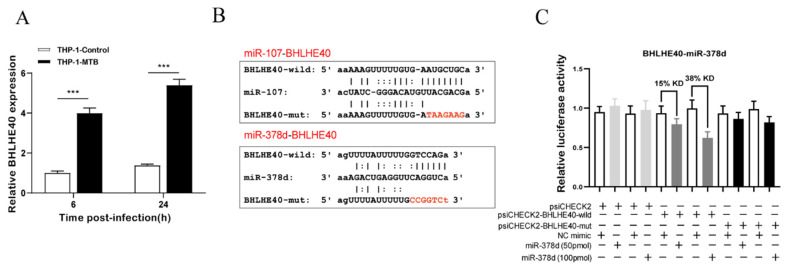
BHLHE40-*miR-378d-BHLHE40* regulation axis verification. (**A**) THP-1 cells were infected with MTB-1458 at a MOI of 10 for 12 h. Cells were washed twice and a medium with gentamycin (100 μg/mL) was added. Thereafter, total RNA was collected at 6 and 24 hpi. *BHLHE40* expression level was evaluated by qRT-PCR. (**B**) The binding site of *miR-378d* with *BHLHE40* 3′-UTR mRNA or mutant of *BHLHE40* 3′-UTR mRNA. (**C**) Dual-luciferase reporter assay was executed in 293T cells. The psiCHECK2, psiCHECK2-*BHLHE40*-wild, and psiCHECK2-*BHLHE40*-mut plasmids were cotransfected with NC mimic or *miR-378d* mimic. The relative luciferase activity means Renilla to firefly ratio (Rluc/Fluc) in miR-378d mimic or NC mimic. KD represents knockdown. “***” represents *p* < 0.001, “+” represents presence, “−” represents absence.

qRT-PCR results indicated that *BHLHE40* mRNA expression was significantly increased in MTB-1458-infected THP-1 macrophages in comparison to uninfected cells and was negatively correlated with the *miR-378d* expression trend (Figure 6A). The interaction between *miR-378d* and *BHLHE40* was identified by the dual-luciferase reporter using psiCHECK-*BHLHE40*-wild and psiCHECK-*BHLHE40*-mut plasmids (Figure 6B). Results showed the Renilla/firefly ratio was significantly lower in *miR-378d* mimic than in negative control (NC) mimic in the psiCHECK2-*BHLHE40*-wild group, and the inhibition effect was dose-dependent (Figure 6C), indicating that *BHLHE40* is the downstream target of *miR-378d*. However, there was no interaction between *miR-107* and *BHLHE40* by the dual-luciferase reporter assay. The expression level of *BHLHE40* and *miR-378d* was negatively correlated during MTB infection and *BHLHE40* was a target of *miR-378d*. However, in-depth investigations are needed to elucidate the detailed regulation mechanisms.

## 3. Discussion

The difference in phenotype induced by virulent and avirulent strains is an effective approach to explore TB pathogenesis. Previous studies showed that, in the virulent MTB strain H37Rv-infected macrophages, 36 genes related to immune response regulation, chemokine secretion, and leucocyte chemotaxis were upregulated, and 30 genes associated with amino acid biosynthetic and energy metabolism, connective tissue development, and extracellular matrix organization were downregulated, compared to avirulent strain-infected macrophage [14]. Virulent *M. bovis* and BCG can also lead to different immune responses associated with TB pathogenesis [2]. This study compared the expression profile of virulent MTB-1458-infected macrophages or avirulent BCG-infected macrophages to uninfected cells. A unique transcriptional network, including TFs and mRNAs of MTB-virulent-strain-mediated DE-miRNAs, was identified to comprehend the pathogenesis of these two strains, which might be favorable to prevent the development of TB.

### 3.1. miRNA Expression Profile Comparison between MTB-1458-Infected Macrophages and BCG-Infected Macrophages

Many studies have highlighted the role of miRNA during MTB infection, including modulation of autophagy, apoptosis, or inflammation, to affect the susceptibility or survival of MTB [15,16,17,18]. In this study, miRNA expression profiles of MTB-1458-infected THP-1, BCG-infected THP-1, and uninfected THP-1 were analyzed by high-throughput sequencing. MTB-1458 induced a stronger miRNA expression profile variation in the entire level compared to BCG-infected macrophages. In all 18 unique DE-miRNAs, only *miR-146a-5p* (*miR-146a*), *miR-378d*, *miR-874-3p* (*miR-874*), and *miR-140-3p* have been reported to modulate the essential biological processes during MTB infection [19,20,21,22,23]. Thus, it is worthwhile to thoroughly research the function of the remaining miRNAs, which would contribute to finding new mechanisms during the interaction between the host and bacteria.

Aside from the DE-miRNAs uniquely induced by MTB-1458, *miR-146a-3p*, *miR-212-3p*, and *miR-132-3p* were differentially expressed in both infection groups. Previous research indicated that *miR-132-3p* (*miR-132*) inhibited IFN-γ expression by targeting *p300*, which was beneficial to the immune evasion of MTB [24]. The “seed” region used for the target prediction of *miR-212-3p* was the same as in *miR-132*, suggesting that they have similar regulatory functions. Moreover, *miR-212-3p* demonstrates upregulation in latent TB infection [25]. However, there are no TB-pathogenesis-related studies about *miR-146a-3p* and *miR-212-3p*. Research shows that *miR-212-3p* is a tumor-associated miRNA, has been proved to influence the vasculogenic mimicry of glioma, and induces autophagy to inhibit osteosarcoma [26,27]. *miR-146a-3p* appears to be a miRNA associated with inflammatory response. Dexmedetomidine functions a cardioprotective effect by manipulating *miR-146a-3p* to inhibit the nuclear factor-κB (NF-κB) pathway [28]. Downregulation *miRNA-146a-3p* suppresses lipopolysaccharide-induced acute lung injury by upregulating *SIRT1* and regulating the NF-κB pathway [29]. Given that they are upregulated during the virulent or avirulent strain infection, studying them may find virulence-related genes of MTB.

The candidate targets for DE-miRNAs were analyzed, and they were mainly enriched in the Foxo signaling pathway (5 of 81), TNF signaling pathway (4 of 81), PI3K/Akt signaling pathway (5 of 81), Rap1 signaling pathway (4 of 81), and C-type lectin receptor signaling pathway (3 of 81). The first four pathways were mainly related to apoptosis, consistent with the result of GO BP. Previous studies have shown that, compared to the avirulent strain, inhibiting cell apoptosis seems to be an effective measure of virulent strain to escape the host’s antibacterial action [30]. Furthermore, C-type lectin receptors were important receptors that could identify pathogen-associated molecular patterns (PAMPs) of MTB. For instance, Mincle recognizes trehalose 6,6′-dimycolate (TDM) to induce proinflammatory factor production [31,32], whereas mannose receptor (MR) is the receptor of ManLAM that facilitates survival of MTB [33,34]. Studying the specific functions of the C-type lectin receptor signaling pathway is conducive for controlling the development of TB by dominating these receptors.

### 3.2. Key DE-miRNAs According to the TF-miRNA-mRNA Network

miRNAs affect the cellular environment and signals by targeting to mRNAs. Meanwhile, their expressions are also regulated by TFs. Therefore, TF, miRNA, and mRNA can form multiple feed-forward and feed-backward loops, providing a novel idea for studying the key role of miRNAs in the disease pathogenesis. TF-miRNA-mRNA regulation has been applied to study cancer pathogenesis and livestock breeding [35,36,37]. This study sought hub miRNAs differentially expressed only in the MTB-1458-infected group and their TFs and mRNA by constructing the TF-miRNA-mRNA network.

The upregulated DE-miRNA network consisting of 5 miRNAs, 12 TFs, and 14 targets was generated (Figure 4A). *miR-146a* has been extensively researched in TB and mediates the immune response by targeting *IRAK-1*, *TRAF-6*, and *PTGS2* [20,38]. However, the mechanism of *miR-146a* upregulated expression is still unclear. These results uncovered that *miR-146a* may be activated by TF-CTNNB1. The function of the remaining four miRNAs in TB has not been studied yet. *miR-335-3p* had the highest node degree, and JUND-*miR-335-3p-MYB/SH3KBP1* was regarded as a key axis in all interactions. JUND is a member of the AP-1 family of TFs and can regulate transcriptional programs of cellular differentiation, proliferation, and apoptosis [39]. SH3KBP1 is implicated in numerous cellular processes, including apoptosis, cell adhesion, and regulation of clathrin-dependent endocytosis. MYB can influence cell survival through the PI3K/Akt signaling pathway. Since apoptosis is one of the differential pathogenic mechanisms during BCG and MTB infection, we suppose *miR-335-3p* may participate in the cell apoptosis process.

The downregulated DE-miRNA network comprised of 13 miRNAs, 11 TFs, and 67 targets (Figure 4B). Three miRNAs (*miR-378d*, *miR-874*, and *miR-140-3p*) have been verified that could play important roles during MTB infection. *miR-3184-5p* had the highest node degree, and MYB-*miR-3184-5p-BMF/IRF1/PML* was regarded as a key axis composed of the higher degree node. Studies demonstrated that *miR-3184-5p* is a tumor suppressor gene that can inhibit cancer cell proliferation [40,41]. In addition, BMF is a member of the Bcl-2 family that is related to apoptosis. IRF1 participates in the IFN-γ and IL-12 signaling pathways. PML is relevant to IFN-γ signaling pathways and SUMOylation. Thus, *miR-3184-5p* may modulate the pathways mentioned above during MTB infection. In addition, some genes are both target and TF; they, hereby, interacted with one miRNA, making it possible to form a feed-forward or feed-backward loop. For instance, *BHLHE40*, *CEBPB*, and *IRF1* all act as vital mediators during pathogen-host interactions [13,42,43]. This network analysis provides directions for further experimental exploration.

The difference of virulence between BCG and MTB can lead to variant immune responses, including immunomodulatory genes, cytokines, and miRNAs. Recently, host-directed therapy (HDT) is considered to be one of the effective ways of treating TB in the future, and the strategies of using miRNA, cytokines, and key immunomodulatory genes as the HDT are being broadly discussed [44,45,46,47]. This study had carried out in-depth data mining of the profiles of miRNAs in THP-1 cells after the virulent and avirulent strain infection and found the key TF/miRNA/mRNA regulation axes by systematic network analysis. The analysis results revealed that cytokines and key immunomodulatory genes related to TB possessed a regulatory relationship with the DE-miRNAs. Therefore, it is reasonable to infer that some of the DE-miRNA can be used for HDT against TB. However, their specific functions need to be validated in a series of subsequent experiments. Moreover, the displayed DE-miRNAs in this research are only based on the THP-1 cell; subsequent verification tests are suggested to be performed with primary macrophages or in vivo. This will help to provide more sound and reliable theoretical knowledge for the development of potential druggable targets for HDT against TB.

In conclusion, this was the first comprehensive TF-miRNA-mRNA network analysis study based on global transcriptome analysis of virulent MTB-infected THP-1 cells or avirulent BCG-infected THP-1 cells and uninfected THP-1 cells. Unique DE-miRNAs in MTB-1458-infected groups as well as their matched targets and TFs were selected to generate the TF-miRNA-mRNA network for excavating key TF, miRNA, mRNAs, and axes. The results provided an excellent suggestion for further understanding the specific immune response between MTB and the host.

## 4. Materials and Methods

### 4.1. Bacterial Strains and Culture

The MTB-1458 (GenBank accession no. CP013475.1) strain was a typical Beijing family strain isolated from a cow and identified in this lab [48,49]. The MTB-1458 and Tokyo BCG (ATCC 35737) strains were grown in Middlebrook 7H9 broth medium (BD, Franklin, NJ, USA), supplemented with 10% oleic acid, albumin, dextrose, and catalase medium (BD), as well as 0.5% glycerol (Sigma-Aldrich, St. Louis, MO, USA) and 0.05% Tween 80 (Sigma-Aldrich).

### 4.2. Cell Culture and Infection

Human acute monocytic leukemia cells (THP-1; ATCC TIB-202) were cultured in RPMI-1640 medium (Hyclone, Logan, UT, USA) with 10% fetal bovine serum (Gibco, Carlsbad, CA, USA) and maintained in a humidified atmosphere at 37°C with 5% CO_2_.

The infection experiment was performed as described previously [50]. Briefly, by using phorbol myristate acetate (40 ng/mL, Sigma-Aldrich) to differentiate THP-1 cells for 12 h. The mid-log phase cultures (MTB-1458 and BCG) were pelleted and resuspended in Hanks’ Balanced Salt Solution (HBSS, Gibco). Bacterial were dispersed into a single-cell suspension by using a 22-gage syringe. The bacterial concentrations (CFU/mL) were estimated by OD_600_ values. The multiplicity of infection (MOI, BCG, or MTB-1458: THP-1 cells) was 10. After 12 h, cells were washed twice to remove extracellular bacteria, and then a medium with gentamycin (100 μg/mL) was added. Thereafter, total RNA was collected at 6 and 24 hpi. There were six groups, including MTB-1458-6 hpi, MTB-1458-24 hpi, BCG-6 hpi, BCG-24 hpi, uninfected-6 hpi, and uninfected-24 hpi. All experimental procedures using live MTB were performed at the Biosafety Level 3 Laboratory at Huazhong Agricultural University.

### 4.3. Total RNA Extraction and Integrity Analysis

Total RNA was isolated using TRIzol reagent (Invitrogen, Carlsbad, CA, USA) in accordance with the instructions of the manufacturer. RNA purity and integrity were checked by agarose gel electrophoresis and quantified by determining the absorbance of A260 on SmartSpec (Bio-Rad, Hercules, CA, USA).

### 4.4. miRNA Profiling by High-Throughput Sequencing

A Balancer NGS Library Preparation Kit for small RNA/miRNA (GnomeGen, San Diego, CA, USA) was used for small RNA cDNA library preparation. In accordance with the instructions of the manufacturer, 3 μg of total RNA was ligated to 3′ and 5′ adaptors, sequentially, reverse transcribed to cDNA and amplified by PCR. Then, 10% natural polyacrylamide gel electrophoresis was used to separate the whole library, as well as cut and elute the bands corresponding to miRNA insertion. The purified small RNA libraries were quantified for cluster generation and 36 nt single-end sequencing analysis using Illumina GAIIx (Illumina, San Diego, CA, USA).

### 4.5. Bioinformatic Analysis

The potential targets of miRNAs were predicted by miRanda [51] and miRDB [52]. The potential TFs of miRNAs were predicted by the TransmiR version 2.0 database [53]. GO classification and KEGG pathway enrichment analysis of the final candidate targets of selected miRNAs were performed in DAVID 6.8 [54] and KONAS 3.0 [55] online database with default settings, respectively. Jvenn was adopted to create the Venn diagram [56]. The associations among miRNAs, mRNAs, and TFs were constructed and visualized through Cytoscape version 3.6.0.

### 4.6. miRNAs Screening Differentially Expressed Only in MTB-1458 Groups

As shown in the flowchart (Figure 2), DE-miRNAs from the BCG-infected groups vs. uninfected groups and DE-miRNAs from MTB-1458-infected groups vs. uninfected groups were selected first. Then, DE-miRNAs only in MTB-1458 groups were selected. Second, DE-miRNAs with a transcript per million (TPM) value of <10 were rejected. Finally, the union of DE-miRNAs upregulated at 6 or 24 hpi, and the intersection of DE-miRNAs downregulated at both 6 and 24 hpi, were further selected.

### 4.7. miRNA and mRNA Expression Validation by qRT-PCR

miRNA and mRNA expression levels were analyzed by qRT-PCR as described previously [50]. Briefly, by using the HiScript II Q Select RT SuperMix kit (Vazyme, Nanjing, China) to reverse transcribe RNA (1 μg) into cDNA. For miRNA, using a miRNA 1st-Strand cDNA Synthesis Kit (GeneCopoeia, Carlsbad, CA, USA) to synthesize cDNA. qRT-PCR were performed with AceQ qPCR SYBR Green Master Mix (Vazyme) in ABI ViiA 7 (Applied Biosystems, Carlsbad, CA, USA). Meanwhile, U6 and β-actin acted as the internal reference, respectively. The 2^−ΔΔCt^ method was adopted to analyze the genes relative expression. Table S2 displayed the related primer sequences.

### 4.8. Identification of the Relationship between BHLHE40 and miR-378d with Dual-Luciferase Reporter Assay

The dual-luciferase reporter assay was performed as described previously [50]. The pairing region with *miR-378d* in partial 3′-untranslated region (3′-UTR) of *BHLHE40* was amplified using the primer pair F, 5′-cctcgagCCAAACCAAGGTCTGAGAAATG-3′ and R, 5′-ttgcggccgcCTCTTCAAAAACAGGAATACATTCA-3′. Overlapping PCR was applied to generate the mutated 3′-UTR with the primer pair of F, 5′-TATTTTTGCCGGTCTGTACTTGTT-3′ and R, 5′-AACAAGTACAGACCGGCAAAAATA-3′.

The mutant and wild-type amplicons were inserted into the psiCHECK-2 vector (Promega, Madison, WI, USA) between the XhoI and NotI restriction sites (psiCHECK-*BHLHE40*-wild and psiCHECK-*BHLHE40*-mut). The psiCHECK-control, psiCHECK-*BHLHE40*-wild, and psiCHECK-*BHLHE40*-mut were cotransfected with the *miR-378d* mimic or NC mimic into HEK293T cells, respectively. After 24 h, the dual-luciferase activity was examined using the Dual-Luciferase Reporter Assay System (Promega).

### 4.9. Statistical Analysis

Data were expressed as mean ± standard deviation. ANOVA was used to analyze the statistical significance of the data by GraphPad Prism (version 8.0). * *p* < 0.05, ** *p* < 0.01, and *** *p* < 0.001 were used to indicate statistically significant differences.

## Figures and Tables

**Table 1:** Clean reads mapping against human mature miRNAs of miRBase.

Group	Input	Total	Unique	Multiple
MTB-1458-6 hpi	2,278,861	799,656 (35.09%)	783,022 (97.92%)	16,634 (2.08%)
BCG-6 hpi	4,949,944	3,517,427 (71.06%)	3,427,613 (97.45%)	89,814 (2.55%)
uninfection-6 hpi	5,370,123	4,449,198 (82.85%)	4,342,620 (97.60%)	106,578 (2.40%)
MTB-1458-24 hpi	2,514,040	843,526 (33.55%)	827,655 (98.12%)	15,871 (1.88%)
BCG-24 hpi	4,561,834	3,332,746 (73.06%)	3,164,199 (94.94%)	168,547 (5.06%)
uninfection-24 hpi	4,271,225	3,251,564 (76.13%)	3,157,811 (97.12%)	93,753 (2.88%)

**Table 2:** The unique DE-miRNAs in MTB-1458 groups and their targets and TFs.

miRNA	Target	TF
Down		
*miR-101-3p*	*ZCCHC2|SGK1|MOB1A|CPEB2|TNPO1|RNF213*	-
*miR-107*	*TNFSF10|ZCCHC2|PALM2-AKAP2|OAS3|NRP2|BHLHE40|TNPO1|PLAGL1*	BHLHE40|CEBPB|HNF4G|IRF1|MYB|PML|RUNX3|TRIM25
*miR-140-3p*	*CMPK2|RGS1|RASGEF1B|HNF4G|SKIL|AFF1|WDFY1|BTG1|RNF213*	ATF3|BHLHE40|CEBPB|JUND|MYB|PML|RUNX3
*miR-146b-3p*	*PML|MOB3C*	ATF3|CEBPB|HNF4G|IRF1|JUND|MYB|RUNX3
*miR-210-3p*	*DHX58|RUNX3|PIK3R5*	BHLHE40|IRF1|JUN|JUND|MYB
*miR-320b*	*CREG1|DTX3L|ZCCHC2|PRKAG2|NT5C3A|PALM2-AKAP2|TMEM123|GNA13|PLK3|ARL8B|CTNNB1|MANBA|BTG1|TNPO1|GPBP1|ZBTB43|GPNMB*	JUN
*miR-378c*	*AFF1|RDX|SRGAP2|ART3|BHLHE40|ARL8B*	-
*miR-378d*	*AFF1|RDX|SRGAP2|ART3|BHLHE40|ARL8B*	ATF3|BHLHE40|IRF1|MYB|RUNX3|TRIM25
*miR-660-5p*	*RDX*	CEBPB|PML|TRIM25
*miR-874-3p*	*FBXO32|PARP14|TXNRD1|TRAF1*	JUN|JUND|MYB|RUNX3
*miR-1261*	*ATF3|SKIL|DAPP1|INSIG1|B2M|SNX10*	-
*miR-3184-5p*	*MRAS|IRF1|SQSTM1|TRIM5|CSF1|TRIM14|PML|SMIM3|FRMD3|BMF|NGFR|CEBPB*	ATF3|BHLHE40|CEBPB|IRF1|JUN|JUND|MYB|PML
*miR-4286*	*ZCCHC2|IRF1|PARP9|MGAT1|ARMC9|TRIM25|INSIG1|TRIM56|BMF|SETDB2*	-
Up		
*miR-1290*	*SDHC|TSC22D1|IGFBP3|RPL36A*	ATF3|BHLHE40|CEBPB|HNF4G|IRF1|JUN|JUND|TRIM25
*miR-96-5p*	*MYB|SH3KBP1|ATG16L1|ADK|NPTX1*	CEBPB|CTNNB1|JUN|JUND
*miR-335-3p*	*MYB|DIAPH1|APEX1|SPN|CPT1A|SH3KBP1|MYO6*	CEBPB|IRF1|JUN|JUND|PML|TRIM25
*miR-625-3p*	*TSC22D1*	BHLHE40|JUND|MYB|PML|RUNX3|TRIM25
*miR-146a-5p*	*-*	BHLHE40|CEBPB|CTNNB1|IRF1|JUN|JUND|MYB|PML|RUNX3

## Data Availability

All data generated in this study are available in the article and its Supplementary Materials.

## Supplementary Materials

The following Supplementary Material are available online https://www.mdpi.com/article/10.3390/ijms23010382/s1.
