# Supplementary material for: Identification of Unique Key miRNAs, TFs, and mRNAs in Virulent MTB Infection Macrophages by Network Analysis
Source: Int J Mol Sci. 2021 Dec 29;23(1):382. doi: 10.3390/ijms23010382 (PMC8745702; doi:10.3390/ijms23010382)
Supplement: Supplementary file 1 [file ijms-23-00382-s001.zip › Supporting Information-Figure.pdf]

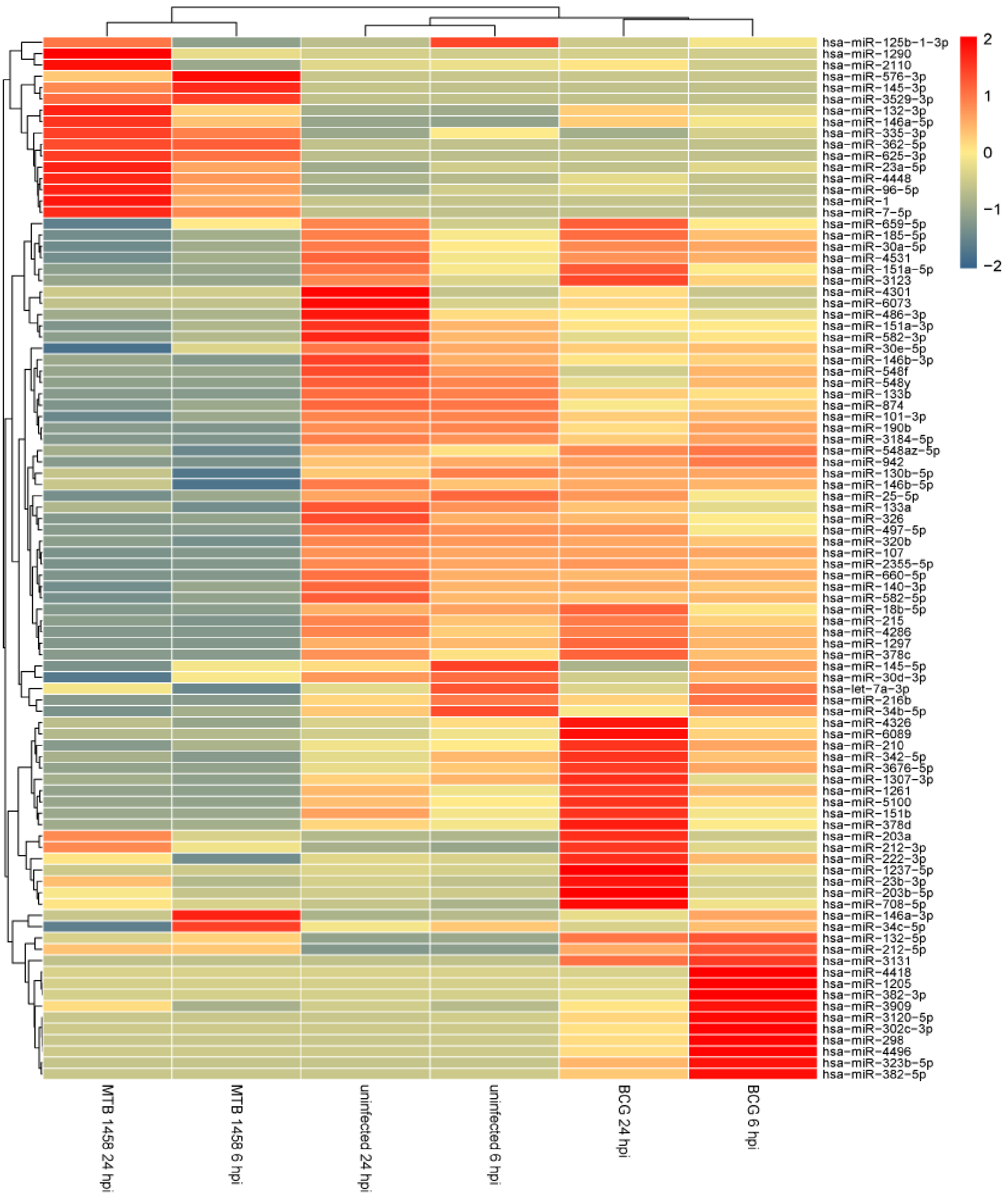

Figure S1. Hierarchical clustering analysis of differentially expressed miRNAs between MTB-1458-infected vs. uninfected cells and BCG-infected vs. uninfected cells

Hierarchical clustering analysis of differentially expressed miRNAs between MTB-1458-infected vs. uninfected cells and BCG-infected vs. uninfected cells. Each row represents miRNA; each column represents a sample. Red indicates higher expression in MTB-1458- or BCG-infected cells in comparison with uninfected cells; blue indicates lower expression in MTB-1458- or BCG-infected cells in comparison with uninfected cells.
